# Cancer immunotherapy by γδ T cells

**DOI:** 10.1126/science.abq7248

**Published:** 2024-10-04

**Authors:** Adrian Hayday, Julie Dechanet-Merville, Jamie Rossjohn, Bruno Silva-Santos

**Affiliations:** 1 Francis Crick Institute, Peter Gorer Dept of Immunobiology, King’s College London, and CRUK City of London Cancer Centre, UK; 2 ImmunoConcEpT, Centre National de la Recherche Scientifique, Unité Mixte de Recherche 5164, University of Bordeaux, Bordeaux, France; 3 Infection and Immunity Program & Department of Biochemistry and Molecular Biology, Biomedicine Discovery Institute, Monash University, Clayton, Victoria, Australia; Institute of Infection and Immunity, Cardiff University, School of Medicine, Heath Park, Cardiff, UK; 4 Instituto de Medicina Molecular João Lobo Antunes, Faculdade de Medicina, Universidade de Lisboa, Lisbon, Portugal

## Abstract

*The premise* of cancer immunotherapy is that cancers are specifically visible to an immune system tolerised to healthy self. *The promise* of cancer immunotherapy is that immune effector mechanisms and immunological memory can jointly eradicate cancers and inoperable metastases and *de facto* vaccinate against recurrence. For some patients with hitherto incurable diseases, including metastatic melanoma, this promise is being realised by game-changing immunotherapies based on αβ T cells. Today’s challenges are to bring benefit to greater numbers of patients of diverse ethnicities, targeting more cancer types, and achieving cure while incurring fewer adverse events. In meeting those challenges, unique benefits may be offered by γδ T cells which compose a second T cell lineage with distinct recognition capabilities and functional traits that bridge innate and adaptive immunity. γδ T cell-based clinical trials, including “off-the-shelf” adoptive cell therapy (ACT) and agonist antibodies are yielding promising results, although identifiable problems remain. In addressing those problems, we advocate that immunotherapies be guided by the distinctive biology of γδ T cells as elucidated by ongoing research.

## Immunotherapies based on Adaptive Immunity

The specific recognition of human cancers and the potential to vaccinate is rooted in adaptive immunity, wherein massively diverse reactivities of cell-type defining antigen receptors, B cell receptors (BCRs / immunoglobulins [Ig]) and T cell receptors (TCRs) derive from the quasi-random somatic recombination of “V-(D)-J” gene segments that encode them, and additionally from somatic mutation for Ig genes(1, 2).

Adaptive immunity in most jawed vertebrates comprises B cells, αβ T cells, and γδ T cells. Contemporary cancer immunotherapy originated in B cell biology, specifically immunoglobulins (a.k.a. antibodies) used to target molecules including EGFR isoforms and CD20 commonly over-expressed by carcinomas and lymphomas, respectively. Such immunoglobulins work in part by ADCC (antibody-dependent cellular cytotoxicity), in which their “Fc” region engages Natural Killer (NK) cells or macrophages *via* “Fc Receptors” (FcR), provoking killing of target cells bound by the antigen-specific Variable (V)-regions of the antibodies. This bridging of adaptive (B cell) and innate (NK cell / macrophage) immunity has been extremely successful, but at least two major limitations exist: intra-tumoral ADCC-competent cells can be scarce(3) and the number of antibody targets discriminating cancer cells from healthy counterparts is *de facto* limited.

These limitations were addressed by using cytolytic T cells which recognise major histocompatibility complex (MHC) proteins (HLA in humans) presenting neoantigenic peptides derived from proteins somatically mutated in cancer cells owing to genome instability(4). However, chronically stimulated, neoantigen-specific, tumour-infiltrating T lymphocytes (TILs) frequently upregulate inhibitory “checkpoint” receptors including PD-1, CTLA-4, TIM3, and LAG3, and become functionally exhausted. Limiting exhaustion by antibody-meditated immune checkpoint blockade (ICB) has enjoyed game-changing success, becoming first-line treatment for multiple solid tumours(5). Nonetheless, many tumours have few mutations and hence few neoantigens, and they can also suppress β2-microglobulin which is required for the expression of Class I MHC-peptide complexes, as well as for both CD1 which presents lipid antigens to TCRαβ^+^ NKT cells, and MR1 which presents metabolite antigens to TCRαβ^+^ MAIT cells(6). Thus, tumours become invisible to a panoply of αβ T cells. Furthermore, ICB-driven αβ T cell derepression *en masse* may induce uncontrolled autoreactivity causing permanent and severe adverse events (AEs)(7).

So-called CAR-T immunotherapy is based on combining B and T cell biology. Specifically, gene segments encoding tumour antigen-specific IgV-regions are fused to gene segments encoding T cell signalling mediators, whereupon the resultant chimaeric antigen receptors (CARs) are introduced *ex vivo* into a patient’s T cells that are expanded and then reinfused(8). By combining antibody specificity with cytolytic T lymphocyte (CTL) capabilities, CAR-T cells deliver ADCC to antigen-expressing cancer cells, invulnerable to MHC loss or low neoantigen load.

Nonetheless, while transformative efficacy has been seen in several haematologic cancers, practical challenges exist(9), including: limited cancer-specific antigenic targets; the fragility of T cells in advanced cancer patients exposed to radiation and/or chemotherapeutics; time-consuming logistics of T cell expansion and transduction; uncertainty that transduction will target CTLs; the likelihood of tumour immune-evasion *via* CAR-specific antigen loss; and the unpredictability of transduced CAR-T cells reaching and thriving within a hostile solid tumour microenvironment (TME), notwithstanding efforts to overcome this by vaccination-based CAR-T boosting(10,11). Additionally, necessary precautions to constrain severe acute AEs limit the numbers of accredited CAR-T treatment centres, each of which is limited in the numbers of treatments it can perform. This raises serious questions about the accessibility and inclusivity of such an expensive treatment.

In sum, the transformative potentials of therapies rooted in adaptive immunity are qualified by the limited spectrum of tumours that they reach, by collateral damage, and by logistics. As will now be considered, γδ-based therapies may be much less affected by such limitations (Table 1).

## γδ cells: Nature’s CAR-T cells

The suitability of γδ T cells for immunotherapy is suggested by their natural CAR-T cell biology. First, γδTCRs function like antibodies in recognising native antigens(12,13), but because TCRδ V-D-J gene-segment recombination may achieve unmatched diversity(12), the range of tumour targets may be substantially broader, including autologous surface antigens expressed at high levels and/or in altered conformations by many different cancers (Table 2). Parenthetically, evolutionary data highlight overlaps of B and γδ T cell biology, with lizards lacking γδ T cells showing B cell amplification, whereas the B cell compartment may be more limited in marsupials harbouring an extra chain, TCRμ, that amplifies γδ TCR diversity(14).

Second, γδ T cells combine antibody-like recognition with: high expression of granzymes and perforin that facilitate target cell lysis(12); some capacity to present antigen to αβ T cells(15); and expression of activating natural killer cell receptors (NKRs) including natural cytotoxicity receptors(16) and Fc-receptors(17, 18) that can supplement TCRγδ-mediated cancer cell recognition. Following nonclonal NKR engagement, γδ T cells can phenocopy innate immune cells in responding rapidly, delivering effector function without prior clonal expansion, and orchestrating adaptive immunity by antigen presentation and by promoting an immunogenic cytokine milieu(19). Likewise, many human γδ T cells are naturally tissue-tropic(12). Nonetheless, human peripheral blood and tissue-associated γδ T cells also phenocopy adaptive immunity in displaying durable clonotypic, TCR-mediated responses to various challenges(20-22). We advocate that this capacity to straddle innate and adaptive immunity endows γδ T cell-based immunotherapies with unique advantages.

## Off-the-shelf therapies

Because γδ T cells are not MHC-restricted, they can be transfused as allografts with little danger of graft-versus-host disease (GVHD) that confounds allogeneic αβ T cell therapies. Hence, in relation to logistics, γδ ACT could be prepared in advance from healthy donors and administered “off-the-shelf”, meeting stringent timeframes for patient treatment, permitting rigorous pre-infusion characterisation and refinement of the product, and permitting the patient to be informed that the identical product has shown demonstrable efficacy in other recipients.

Nonetheless, allogeneic grafts risk rejection by histo-incompatible hosts(23). Several “cloaking” approaches have been developed to limit this, most often by reducing MHC expression, which is particularly facile for γδ ACT derived from inducible pluripotent stem cells(24). Alternatively, CAR-T cells can be transduced with an alloimmune defense receptor (ADR) comprising part of the ligand for 4-1BB linked to a CD3 signalling motif(23). Since alloreactive T and NK cells disproportionately upregulate 4-1BB, they can be specifically targeted by ADR-expressing γδ CAR-T cells that thereby escape deletion. Additionally, a “veto” effect exists whereby adoptively transferred NK cells target graft-reactive CD8 T cells(25): given their parallels with NK cell biology, γδ-based ACT might be optimised to veto their rejection.

## Natural cancer cell recognition by blood Vγ9Vδ2 T cells

γδ-based immunotherapies have primarily focussed on Vγ9Vδ2 T cells, the predominant blood γδ T cell subset, and Vδ1 T cells that are commonly enriched in tissues. Being easier to obtain, Vγ9Vδ2 T cells were first into the clinic(26). Vγ9Vδ2 T cells rapidly respond to myriad microbial infections, commonly reflecting polyclonal TCRVγ9Vδ2 reactivity to “phosphoantigens” (pAgs), hydroxymethy-but-2-enyl pyrophosphate (HMBPP) and isopentenyl pyrophosphate (IPP). HMBPP is an intermediate in the microbial MEP (methylerythritol phosphate) pathway that generates cholesterol and sterol derivatives, whereas IPP is an intermediate common to the MEP pathway and its host cell counterpart, the mevalonate pathway(27). Virus-infected and cancer cells often upregulate IPP, e.g., by hydroxy methyl glutaryl co-enzyme reductase upregulation. pAgs bind the intracellular B30.2 domain of Butyrophilin 3A1(BTN3A1)(28), cementing association with BTN2A1 which directly binds TCRVγ9(29–32). BTN and BTN-like (BTNL) proteins are understudied Ig-domain-containing members of the B7-superfamily of lymphocyte regulators(33). Hence, rather than detecting pathogen-specific or cancer cell-specific antigens, Vγ9Vδ2 T cells recognise altered surface expression of BTN2A1/3A1 as immediate consequences of infection or cell transformation(12) and of AMPK sensing of ATP levels during metabolic crisis in cancer cells(34).

BTN2A1 binds to germline-encoded residues of TCRVγ9, eliciting nonclonal responses that are defining hallmarks of innate immunity(35,36). Nonetheless, CDR3δ sequences are also important, implying that additional TCR contacts are made, possibly with BTN3A1 and/or its relatives, BTN3A2 and BTN3A3, which are required for optimal pAg responses(37–39). Vγ9Vδ2 TCRs are not conserved in rodents which has inevitably limited the preclinical models available to support the development of Vγ9Vδ2 T cell-based immunotherapeutics(40).

## Immunotherapeutic Vγ9Vδ2 T cells

Natural and synthetic pAgs, e.g., the drug BrHPP (Phosphostim®), can support Vγ9Vδ2 T cell expansion *in vitro* as a preliminary to ACT, but they display poor pharmacokinetics *in vivo*. Instead, intracellular IPP levels can be elevated by amino bisphosphonates (ABPs) which inhibit farnesyl pyrophosphate synthase that catalyses geranyl pyrophosphate catabolism downstream of IPP in the MEP and mevalonate pathways(40,41). Because ABPs, e.g., zoledronate and pamidronate, were clinically approved for treating osteoporosis and /or osteolytic cancer metastases, it was easier to obtain regulatory approval for them as Vγ9Vδ2 cell activators in cancer settings. While largely safe, these approaches showed limited clinical efficacy, commonly attributed to Vγ9Vδ2 cell exhaustion caused by chronic stimulation.

These disappointments notwithstanding, Vγ9Vδ2 T cell-based immunotherapeutics remain attractive for many reasons considered above and listed in Table 1. Moreover, ICB combination-therapy has the potential to derepress Vγ9Vδ2 T cells(42). In a Phase I trial of allogeneic Vγ9Vδ2 T cells at Fuda Cancer Hospital, China, safety was confirmed and 18 patients with advanced liver or lung cancer receiving five or more infusions showed greatly prolonged survival (Table 3). In8Bio (Birmingham, USA) has likewise developed Vγ9Vδ2 cells for allogeneic treatment of leukaemia following haematopoietic stem cell transplantation (HSCT) (Table 3). The logic is based on many years’ findings that when risk of relapse was high post-HSCT, long term clinical remission positively correlated with robust and durable γδ T cell reconstitution(43, 44). Preliminary results appear promising, including no current reports of GVHD, durable Vγ9Vδ2 T cell reconstitution probably attributable to highly effective lymphodepletion pre-infusion, and disease stabilisation.

In8Bio has also developed an innovative protocol termed drug resistant immunotherapy (DRI) that focusses the innate responsiveness of Vγ9Vδ2 T cells toward chemotherapy-treated tumours *in situ*. Specifically, Vγ9Vδ2 cells from glioblastoma (GBM) patients are expanded using zoledronate + IL2, whereupon the cells are transduced with a methylguanine DNA methyltransferase (MGMT) gene that confers resistance to temozolomide (TMZ), a chemotherapeutic standard-of-care for GBM, that by inducting genome damage may induce TCR and NKR antigens for Vγ9Vδ2 cells. The MGMT-transduced Vγ9Vδ2 cells are then administered proximal to the residual tumour site *via* a Rickham catheter used for TMZ delivery. Improved methods for Vγ9Vδ2 cell preparation and maintenance offer opportunities to repeatedly and locally administer fresh, non-exhausted cells. DRI was successfully applied to four human/mouse xenograft models of primary and refractory GBM(45) and is being delivered clinically (Table 3).

In parallel to ACT, antibody-based engagers are being developed to activate and expand Vγ9Vδ2 cells *in vivo*, thereby countering the cells’ presumed exhaustion in the TME. Because their numbers are limited, Vγ9Vδ2 activation *en masse* is less likely than αβ T cell agonism to cause AEs. Based on the role of BTN3A molecules in Vγ9Vδ2 T cell activation, ImCheck Therapeutics (Marseille, France) has developed BTN3A-specific agonist antibodies that substitute for pAgs in driving TCR acitvation. With evidence for tumour suppression in xenograft models reconstituted with Vγ9Vδ2 T cells(46), an antibody (ICT0) has been in a phase 1/2a clinical trial in haematological and solid cancers (Table 3), also with promising results.

Likewise, Lava Therapeutics (Utrecht, The Netherlands) has developed bispecific antibodies, so-called “gammabodies” combining tumour-targeting specificities with a TCR Vγ9 binding domain(47). Those reagents activated Vγ9Vδ2-dependent cytotoxicity against tumour cells *in vitro* and based on encouraging pre-clinical data, clinical trials commenced. Whereas Lava discontinued (albeit not for safety reasons) a Phase I trial in haematological cancers of LAVA-051 that co-jointly targeted Vγ9 and CD1d, there is a Phase I trial in metastatic castration-resistant prostate cancer, using LAVA-1207 that co-jointly targets Vγ9 and PMSA (Table 3). Other targets include CD123 and CD40 for blood cancers, EGFR for solid tumours, and undisclosed targets in partnership with Janssen (Pennsylvania, USA). γδ-engagers may be insufficient to fully overcome γδ T cell suppression by the TME, but might be effectively combined with ICB modalities targeting checkpoints most relevant for Vγ9Vδ2 cells.

Combining ACT and engagers, Acepodia (California, USA; Taipei, Taiwan) has used innovative chemistry to conjugate allogeneic Vδ2^+^ T cells to anti-CD20 (ACE1831), for treatment of non-Hodgkin’s lymphoma (Table 3). Encouraging safety and efficacy data reported in May 2024 have added momentum to an analogous approach (ACE2016) targeting EGFR-expressing solid tumors.

There have been multiple uses of Vγ9Vδ2 cells as CAR-T cells. For example, those targeting MUC1-Tn showed similar or superior potency to CAR-αβ T cells *in vitro*(48), and could be sustained *in vivo* with human cytokines, displaying IL-2-dependent activity against a metastatic gastric cancer cell line. The attractiveness of CAR-Vγ9Vδ2 T cells should be enhanced by culture methods that improve cell yields and cytolytic potentials, as reported by Leucid (London, U.K.)(49). Another attractiveness is their overcoming off-target activity and exhaustion attributable to high background phosphoprotein levels induced in αβ T cells by CD3ζ-based CARs, a phenotype not observed in Vγ9Vδ2 T cells transduced with chimaeric co-stimulatory receptors that signal *via* DAP10 and PI3-kinase thereby synergising with CD3ζ-signals induced *via* the natural Vγ9Vδ2 TCR(50). Nevertheless, it will be important to ascertain that potent CAR-driven signalling does not disrupt signature, innate-like, γδ T cell responses to cytokines and NKR ligands.

Avoiding such concerns, and because γδ T cell numbers can be very limiting, Gadeta (Utrecht, Netherlands) combined Vγ9Vδ2-mediated cancer cell recognition with proven CAR-T cell modalities, by transducing primary αβ T cells with Vγ9Vδ2 TCRs selected for relatively high affinity and strong tumour cell killing(51–53). The resulting TEG (T Cells Engineered to Express a Defined Gamma Delta TCR) have been applied in phase I as ACT targeting multiple myeloma (Table 3). Likewise, Immunocore (Oxfordshire, UK) is developing soluble “ImmTAC” constructs, combining Vγ9Vδ2 ectodomains with an anti-CD3 domain, thereby eliciting substantial T cell responses toward cells recognised by TCRVγ9Vδ2. Lacking MHC-restriction, Vγ9Vδ2-ImmTACs may be efficacious in greater numbers of patients than MHC-restricted TCRαβ-based ImmTacs in clinical use(54), but the potential for AEs will need scrutiny.

## Natural cancer cell recognition by non-Vγ9Vδ2 γδ T cells

As well as Vγ9Vδ2 T cells, human blood contains Vδ1^+^, Vδ3^+^, and Vδ5^+^ T cells that are usually strikingly enriched in tissues(55–58). Vδ1^+^ cells are the most abundant and have therefore received most attention, but because their biology seems largely applicable to Vδ3^+^ and Vδ5^+^, it is common to refer to these cells collectively as “non-Vγ9Vδ2” or “Vδ2^neg^” γδ T cells. Of note, repertoire deep-sequencing from blood and tissues has often revealed large, durable, clonal expansions of Vδ2^neg^ cells which are rarely shared across individuals(20–22), implying some form of immunological memory that is a hallmark of adaptive immunity highly describable in immunotherapy. However, the molecular basis of such “γδ memory” remains unelucidated.

**Fig. 1:**
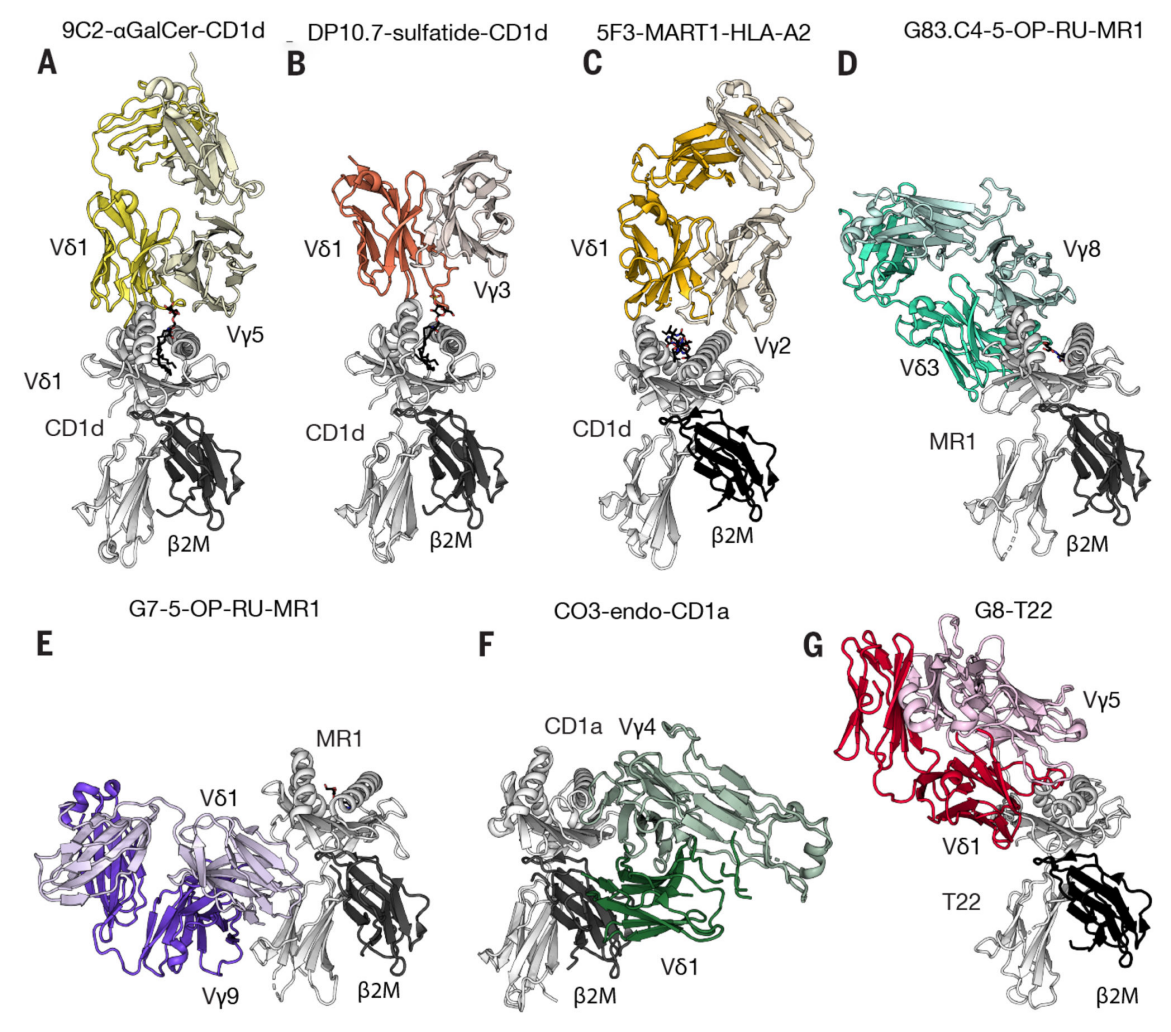
Gallery of human γδTCR structures solved in complex with their ligands highlighting diverse docking modes **(a)** Vγ5Vδ1 TCR-CD1d-α-GalCer **(b)** Vγ3Vδ1 TCR-CD1d-sulfatide **(c)** Vγ2Vδ1 TCR-HLA-A2-MART1 **(d)** VγδVδ3 TCR-MR1-5-OP-RU **(e)** Vγ9Vδ1 TCR-MR1-5-OP-RU (**f**) Vγ4Vδ1 TCR-CD1a-endo (**g**) Vγ5Vδ1 TCR-T22. MHC and MHC-like molecules coloured in grey; distinct coloring for the γδTCRs.

Candidate ligand approaches have identified some overlaps of Vδ1 and αβ T cell reactivities (Table 2), including NKT-like reactivities toward CD1d(59–61). Interestingly, CD1d-restricted Vδ1 T cells can be found within human hepatosplenic T cell lymphomas that can be very aggressive(62). There are also Vδ1 and Vδ3 TCR reactivities toward other CD1 molecules(63–66), which can be over-expressed on haematological malignancies, and towards MR1 (Fig. 1)(67), and Vδ1 T cells recognizing melanoma-associated antigens presented by MHC could be generated *in vitro* from haematopoietic progenitors(68).

Nonetheless, γδTCR and αβTCR reactivities are distinct. Thus, while some lipids (e.g., sulfatides) can increase Vδ1^+^ cell recognition of CD1d(59), they are not mandatory(69). Likewise, whereas αβTCRs bind MHC, CD1 and MR1 ligands in ‘end-to-end’ orientations, the antibody-like nature of γδTCRs is evident in ‘down under’ and ‘sideways’ recognition modes(66,67) (Fig. 1). Some such γδTCRs display reasonable affinities for ligands but nevertheless signal poorly, conceivably attributable to unusual docking modes akin to how the TCRαβ-pMHC docking orientation affects CD8^+^ T cell signalling(70). This underscores how constraining it may be to view γδ T cells simply as αβ T cells with unconventional TCRs: rather, the cells’ optimal clinical exploitation will rely on elucidating exactly how TCRγδ-ligand engagement transduces signals, particularly when integrated with other inputs, e.g., NKRs and cytokines, thereby dictating consequent cell expansion, homing, effector function, and durability.

In this regard, unbiased methods have identified novel tumour antigens recognized by non-Vγ9Vδ2 T cells (Table 2). For example, clonally expanded Vδ1, Vδ3 and Vδ5 T cells from immunosuppressed patients showing CMV reactivation, displayed dual reactivity toward CMV-infected and tumour cells, and by immunizing mice with the target tumour cells, antibodies were obtained that blocked clonotypic tumour cell killing. This identified relevant TCR ligands including the tyrosine receptor EphA2, a membrane translocated form of Annexin A2, and Endothelial Protein C Receptor (EPCR) which is a CD1d homolog(71–73). Each was recognized in native conformation, and each was over-expressed on several cancer cell types and on cells dysregulated by AMPK activation or oxidative stress.

In another approach, CRISPR/Cas9 deployment identified HLA-DR as a ligand of a γδTCR expanded in the context of CMV infection and showing reactivity toward B cell lymphomas(74). Likewise, there are data for TCRγδ recognition of unusual MHC conformations not commonly found on healthy cells (J.D.-M., unpublished). It is intriguing that despite the potential for diversity in TCRδ, many ligands show core structural relatedness to MHC, but with no evidence of tumour-specific antigenic cargoes. Provocatively, Kaufman speculated that hereditary Vγ/Vδ selection may have been based on MHC-like “W” genes, since been lost from most vertebrates(75,76). Additionally, many nonpolymorphic MHC molecules lacking ascribed functions might have fundamental roles as “stress antigens” underpinning γδ T cell-dependent, neoantigen-independent immunosurveillance(77).

Going forward, unbiased TCRγδ antigen identification approaches, including use of TCRγδ multimers, will be coupled with spatial “-omics” to evaluate antigen expression in tumours and assess correlations with clinical outcomes. These direct, practicable methods can complement complex yet evolving approaches to predict HLA-dependent cancer neoantigens for αβ TILs(4). Of note, the unprocessed nature of γδ tumour antigens makes them relatively easy to target *via* “binders” comprising either γδ TCRs or monoclonal antibodies blanketing epitopes on the same targets. Additionally, the overlap of TCRγδ ligand expression in cancers and infections, e.g., CMV(20,78) or toxoplasmosis(79), facilitates studies of tumour-reactive γδ T cells in patients without the complexities of immuno-toxic cancer treatment regimens.

## Immunotherapeutic non-Vγ9Vδ2 T cells

The application of Vδ1^+^ cells to cancer immunotherapy (Table 3) builds on strong associations between the numbers and activation state of Vδ1^+^ cells and progression-free and overall survival in haematological and solid cancers, including breast, lung, and colorectal carcinoma (CRC)(80–82). In CRC liver metastases, Vδ1 T cells constituted the largest TIL subset, showed potent Type 1 effector functions, and correlated with lower metastasis numbers and improved overall survival(83). Likewise, associations of γδ T cell reconstitution and survival post HSCT (above) were stronger for blood Vδ1^+^ cells than for Vδ2^+^ cells(43).

Vδ1 ACT was enabled by clinical-grade protocols for robust cell expansion from tissues or from blood(84). In particular, the “Delta One T (DOT)” protocol achieves >1,000-fold expansions in 2-3 weeks, while also inducing upregulation of NKRs contributing to tumour cell targeting(84, 85). DOT cells showed marked efficacy in various patient-derived xenograft models of AML(85, 86), an indication for which the cells have been granted orphan drug designation for allogeneic application by Takeda Pharmaceuticals (Boston, USA). In an alternative approach, Onechain Therapeutics (Barcelona, Spain) uses Notch-activated CD34^+^ stem cells as its source of allogeneic Vδ1 ACT.

To date however, there has been little exploitation of the cells’ adaptive biology, leaving scope for therapeutic optimisation, e.g., by elucidating the basis of durable clonotypic responses to cancer-associated antigens considered above. The limited polymorphisms of such antigens, e.g., EPCR, suggests a potentially broad-ranging utility, for instance by coupling cancer-targeting Vδ1 TCRs to cytotoxic drugs, evoking antibody-drug conjugates (ADC) that are showing immunotherapeutic successes(87). Currently there are no clinical data from the use of engagers to specifically activate non-Vγ9Vδ2 T cells.

By contrast, cells expanded by the DOT or similar approaches have proved amenable to CAR engineering, e.g., targeting CD123(86), CD20(88), or glypican-3(89). Independently conducted studies demonstrated that CAR-Vδ1 T cells were extremely potent *in vitro* and *in vivo*, while also revealing the importance of human IL-15 for persistence in immunodeficient murine hosts(87, 89).

Of note, allogeneic CD20 CAR-Vδ1 T cells developed by Adicet Bio (Boston, USA) showed favourable interim results in a phase 1 trial in B cell malignancies, including those failing αβ-CAR-T therapy (Table 3), although long-term efficacy will likely depend upon solving “the durability problem”, i.e., to sustain donor cells post-infusion. Recently, Adicet was granted Phase I trial approval for ADI-270 comprising allogeneic Vδ1 cells expressing both a signalling CD27 CAR that binds CD70 overexpressed on renal cell carcinoma, and a dominant negative TGFβ receptor to limit immunosuppression by the TME.

Hedging their bets, Luminary Therapeutics (Minneapolis, USA) use allogeneic mixtures of Vδ1^+^ and Vδ2^+^ T cells as substrates for non-viral delivery of large genetic payloads that cloak the cells (see above) and that targets multiple solid tumor antigens, e.g., BAFF receptors overexpressed on B cell malignancies (Table 3), and CD70 and CSPG4 overexpressed in head-and-neck cancers.

## Immune checkpoint blockade, γδ competence, and tissue normality-sensing

The expanding armamentarium of γδ-based therapeutics needs to be viewed in context. Indeed, whereas ICB is largely viewed as derepressing αβ T cells in the TME, Vδ1 T cells were recently invoked to explain the efficacy of anti-PD-1 in MHCI-deficient CRC(90, 91). Likewise, positive outcomes of ICB in melanomas with low mutational burden (LMB) were significantly associated with high Vδ1 transcript levels(92). Such data offer frontline evidence that γδ T cells can broaden the range of tumours targeted by αβ T cells and extend the reach of ICB, which is important given that early applications of γδ-based immunotherapies in solid tumours will likely need to be in combination with standard-of-care ICB. Vδ1 T cell efficacy in MHC^low^ CRC and LMB melanoma, together with studies of Vδ2^neg^ T cells in kidney cancer(93) seem consistent with evidence that PD-1^+^ Vδ1 cells do not comply with the gene expression signature of exhausted αβ T cells, and very rapidly generate potent effector responses upon derepression(92).

Conceivably PD-1 contributes to controlling tissue-resident γδ T cell activation at steady-state. Indeed, healthy human colonocytes are not ignored by TCRγδ^+^ intestinal intraepithelial lymphocytes (IEL) but are engaged *via* TCR binding to BTNL3+BTNL8 dimers(58). BTNL3 binds germline-encoded Vγ4 sequences within or abutting CDR2 and Hypervariable region (HV)4. This innate modality, which contrasts with CDR3 motifs that confer clonotypic adaptive specificities(64, 94), was subsequently shown for Vγ9-BTN2A1 binding(31,38) (above), and probably underscores Vγ7-BTNL6 engagement in the mouse small intestine where healthy enterocytes express BTNL6-BTNL1 dimers(64). Neither *Btnl1*-deficient mice nor humans hypomorphic for *BTNL3* develop normal TCRγδ^+^ IEL repertoires (58,95), and likewise skin TCRVγ5^+^ IEL fail to mature in mice lacking SKINT1 or SKINT2, two BTNL-related proteins expressed by foetal thymic epithelial cells and keratinocytes(96).

Importantly, however, steady-state TCR-BTNL interactions have profound impacts beyond γδTCR repertoire selection. Thus, in mice transiently exposed to blocking anti-SKINT1 antibodies, the differentiation programme and viability of keratinocytes were compromised resulting in impaired epidermal barrier function. Additionally, intraepidermal γδ cells lost competence to respond to local challenges including ultraviolet irradiation, with consequent accumulation of mutagenic cyclobutane pyrimidine dimers and local inflammatory lesions(97).

The active engagement of healthy tissues by local γδ T cells is termed “normality sensing”(97), and has distinct implications for cancer therapy. First, signal transduction from innate TCRγδ engagement evidently promotes effector functions distinct from cytolysis and inflammatory cytokines induced by adaptive ligands. Second, TCRγδ agonists mimicking innate engagement might sustain γδ T cell competence, potentially solving “the durability problem” (above). This might likewise be achieved by an appropriately designed TCRγδ-based CAR. Indeed, normality sensing conferred competence to respond *via* the 4-1BB (TNFRSF9) co-stimulatory receptor(97), the signalling domain of which is commonly included in latter generation CAR constructs. Conversely, over-active CAR-T signalling might promote exhaustion by overriding innate TCR signals that maintain the cells’ competence. Third, normality sensing γδ T cells can contribute to tissue integrity and to limiting inflammation probably related to the cells’ capacity for wound healing (98,99). This might usefully promote cancer lesion resolution at distinct stages of treatment, e.g., post-surgery adjuvant settings. Improved models to test this hypothesis seem merited given that cancers are described as “wounds that do not heal”(100). Fourth, by actively discriminating normal cells from cancer cells by use of innate and adaptive modalities, γδTCRs naturally create a therapeutic window limiting attacks on healthy tissues.

## Therapeutic windows and rethinking activation thresholds

**Fig. 2:**
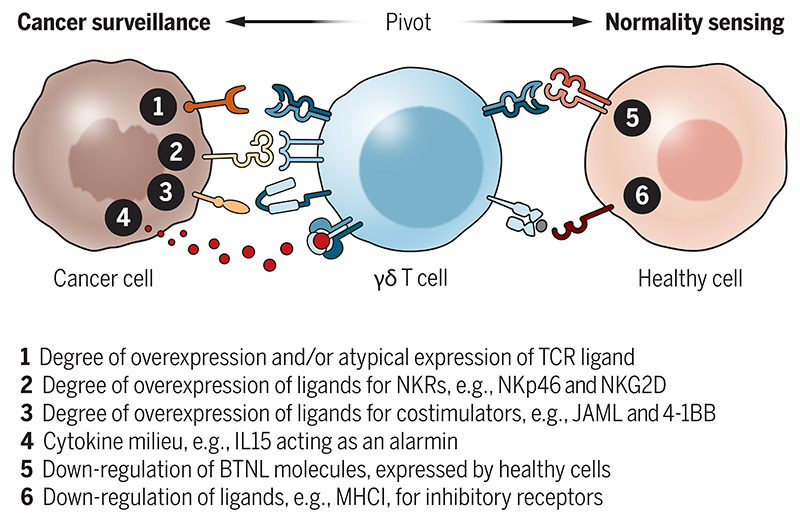
A multipartite-avidity model to maintain peripheral tolerance to self, thereby creating a therapeutic window. A γδ T cell can pivot from normality sensing to cancer cell surveillance by loss of healthy cell markers and acquisition of TCR and innate receptor ligands, a combination of which sets the threshold for full cell activation.

For immunotherapies, the therapeutic window equates to tolerance of normal self. This would seem particularly germane to γδ T cells given their focus on self-antigens. Biochemical and genetic data are consistent with deletion of developing γδ T cells carrying high affinity TCRs, or with their phenotypic skewing away from IL-17(12). Additionally, tolerance may be imposed peripherally by several thresholds limiting γδ T cell activation (12) (Fig. 2). The first would be high ligand density, which contrasts with the capacity of TCRαβ to initiate responses to very low pMHC densities(101), facilitated in part by TCR-ligand catch-bonds that TCRγδ cannot form(102). Indeed, the αβ T cell immunotherapy paradigm of “higher affinity is better” may not apply to γδ T cells if enhanced affinity γδTCRs adversely target normal cells expressing low levels of cognate ligands.

The next class of thresholds (Fig. 2; numbers 2-4) would be overexpression by cancer cells of ligands for different classes of innate receptors including: (i) co-stimulators not commonly implicated in αβ T cell activation, e.g., JAML, which binds the coxsackie-adenovirus entry receptor(103), and CD100, which binds plexin B2(104); (ii) cytokine receptors, e.g., IL-15R that can detect high levels of IL-15 as an alarmin; (iii) scavenger receptors among which the most thoroughly explored γδ T cell activator is WC1 expressed by *Artiodactyla*(12,105); and (iv) NKRs, e.g., NKp46 which recognises ecto-calreticulin induced by endoplasmic reticulum stress that is common in cancer cells(106), and NKG2D, which binds self-encoded ligands induced by DNA damage, excessive growth factor receptor signalling, osmotic shock, and other perturbations(16). Applying this knowledge, Vγ9Vδ2 cells have been transduced with a CAR with the ectodomain of NKG2D, promoting their targeting of solid tumours expressing NKG2D ligands(107). Based on positive pre-clinical results in a xenograft ovarian cancer model, this approach has reached clinical testing in relapsed or refractory solid cancers (Table 3). Similarly, NKp46 was a defining trait of anti-tumour human Vδ1 T cells in colorectal cancer(82), and likewise of BTNL3-selected colonic γδ IEL that seemingly limit IBD severity(95), a predisposing condition for CRC.

Further thresholds would be downregulation of ligands, including BTNLs that sustain γδ T cells rather than fully activate them (above), and MHCI that could suppress γδ T cells by signalling from killer inhibitory receptors (KIRs)(108) (Fig. 2). Which of these (and possibly other) thresholds underpins natural target cell discrimination by γδ T cells in different cancer types needs to be determined if γδ T cell-based therapies are to be optimised in a timely fashion. Importantly, the clear phenotypic distinction of competent *versus* functionally differentiated *versus* exhausted *versus* stem-like progenitor γδ T cells in normal tissues and tumours should provide invaluable prognostic biomarkers for the success or otherwise of γδ T cell immunotherapies. Spatial “-omics” will be essential, as will a better understanding of how γδ T cell signalling integrates innate and clonotypic inputs to promote different phenotypic outcomes.

## Achieving appropriate immunotherapeutic phenotypes

In addition to maintaining appropriate therapeutic windows, the community needs to guard against potentially adverse γδ phenotypes. γδ T cell deficient mice are significantly more susceptible in multiple solid tumour models(109), and mice expressing a Vγ1Cγ4 TCR transgene showed increased resistance to T cell lymphomas(110). Added to this are several aforementioned associations of γδ T cells with favourable outcomes in human cancer(111). Nonetheless, cancer promotion has been ascribed to innate IL-17-producing γδ cells, commonly found in subepithelial tissues of lung, skin, reproductive organs and other sites, where they are readily activated by IL-1β and IL-23(109). Moreover, there have been some associations of γδ T cell activation and worse clinical outcomes, including in pancreatic cancer(111, 112). Toward resolving these paradoxes, elegant mouse molecular genetic experiments demonstrated that TCRγδ^+^ IEL, including BTNL-selected NKp46^+^ cells protected against early stages of CRC, whereas if such control was evaded, invasive CRC growth was enhanced by IL-17-producing γδ cells(113). Thus, it has become commonplace to screen against IL-17 production in evaluating clinical protocols for γδ T cell activation in ACT or *via* engagers. Nonetheless, human IL-17-producing γδ T cells have been extremely challenging to identify or induce(95) and a large-scale study showed negligible *IL-17* RNA expression by human γδ T cells in CRC(114).

Interestingly, whereas the cytolytic activity of TCRαβ^+^ CD8 T cells is a lynchpin of cancer immunotherapies, there are murine tumour-promoting (“T-pro”) CD8 T cells that combine IL-17 production with other traits, including amphiregulin production(115). Similarly, amphiregulin-producing Vδ1 T cells identified in CRC were distinct from CRC-associated cytolytic Vδ1 cells(116). This has fuelled γδ T cell culture protocols skewed against amphiregulin production. Nonetheless, amphiregulin-producing murine γδ T cells are strongly associated with tissue repair(12), again raising the possibility that amphiregulin-producing human cells might be beneficial in driving wound resolution at appropriate treatment junctures.

## Toward cancer remissions; durable immune orchestration

There has been much debate over whether the most critical trait of CAR-T cells is their initial impact on a tumour or their long-term sustainability that permits durable immune surveillance. As has been considered, γδ T cells can satisfy both demands by combining rapid, nonclonal delivery of effector functions with durable clonotypic expansions. Thus, a major practical goal is for γδ–based therapeutics to deliver this combination, with one intriguing possibility being to directly gene-edit endogenous γδ T cells *in vivo*.

Of note, γδ T cell immunotherapies may achieve sustainable cancer remissions *via* innate orchestration of true adaptive immunity. For example, γδ T cells are prominent and beneficial in early responses of cattle to vaccination(12), and human γδ T cell representation strongly correlated with malaria sporozoite vaccination immunogenicity(12,117,118), as may also be true in emerging cancer vaccine settings. Indeed, establishment of high quality CD8^+^ TCRαβ^+^ memory cells was γδ T cell-dependent in settings as diverse as West Nile virus infection(119) and contact hypersensitivity(120), and may also be so in cancer. Thus, γδ-based immunotherapies should not be viewed merely as filling gaps where αβ T cells are ineffective, but as adjuvants that promote αβ T cell efficacy. This re-emphasises that off-the-shelf, allogeneic γδ T cell-based immunotherapies should most likely retain innate competences that swiftly reboot host adaptive immunity, prior to γδ ACT graft rejection.

## Concluding remarks

The distinctive capacity of γδ T cells to bridge innate and adaptive immunity makes them highly attractive candidates for tackling cancer. γδ T cell immunotherapeutics are in clinical trials, reflecting practical advances in expanding and engineering cells for ACT and in developing γδ T cell-specific engagers. Safety profiles seem good, and there are promising read-outs of efficacy. Furthermore, γδ-based immunotherapies are predicted to combine well with ICB in solid tumours, emphasising the need for reliable markers of γδ T cell status, particularly within tumors.

Good science underlies good drugs, and the refinement of first generation γδ-based therapies will benefit from instilling “γδ-unique” traits that in turn require advances in better understanding γδ T cell biology. Ideally, this will integrate efforts in basic research, experimental medicine, pharmaceutical science, and trial design. The goals are cheaper, off-the-shelf treatments that benefit increased numbers of patients of diverse ethnicities with a wider range of cancers; fewer AEs; and reliable prognostic biomarkers of treatment success. As we move beyond some of the constraints of αβ T cells, we should learn how factors in the TME specifically suppress γδ T cells, thereby identifying additional therapeutic targets. Current research efforts are intensive and there is good reason for optimism.

## Figures and Tables

**Table 1:** γδ T cells offer escape from issues limiting αβ T cell-based immunotherapies

Phenotype relevant to immunotherapies	αβ T cells	γδ T cells
MHC-restriction limits unrestricted clinical application	Yes, for most αβ T cells	No obligate MHC restriction for γδ T cells
Resistant to cancer cell loss of β2-microglobulin	No	Yes (function may be enhanced by this status) Yes: no evidence for widespread GVHD
Function as allogeneic therapy off-the-shelf (a)	No: drives graft-versus-host disease (GVDH)	
Function as allogeneic therapy off-the-shelf (b)	No: requires "cloaking" to avoid rejection	No: requires "cloaking" to avoid rejection; may also veto rejection
Home to and function within extralymphoid tissues	Some subsets (e.g., T_RM_) adapt to tissues	Many subsets naturally localize to and function within tissues
Readily recognise tumors with low neoantigen load	Only unconventional subsets (NKT, MAIT) may do this	Yes
Recognise a potentially vast diversity of cancer surface antigens	No	Yes
Responds to ICB	Yes	Yes, with PD-1+ non-Vδ2 cells showing less exhaustion than PD1+ CD8+ αβ T cells
Mostly cytolytic	No	Yes
Adverse events	Potentially high because of CRS and cross-reactivity to normal self	Limited CRS because small fraction of CD3^+^ cells, and because of natural therapeutic window
Establishing cure via immunological memory	Yes, directly	Yes, with capacity to orchestrate CD8+ αβ T cell memory
ADCC	No, unless CAR-T engineered	Yes, naturally
Capacity to cross-present peptide antigens to αβ T cells	No	Yes for Vγ9Vδ2 T cells

**Table 2:** Ligands of human γδ TCRs for which direct binding is documented

Ligand	TCRγδ V-usage	Origin of γδ T cells, context	Affinity (K_D_)	Structure of the TCR/ligand complex	References
**HLA or HLA-like**					
**CD1a**	**Vδ1/Vγ4**	**PBMC-sorted** **γδ T cells using** **CD1a tetramers**	**15-24 μM**	**resolved**	**Wegrecki, Nat Commun.** **2022, PMID: 35790773**
**CD1b**	**Vδ1**	**PBMC-sorted γδ T cells using** **CD1b tetramers loaded with microbial lipids**	**9 μM**	**not reported**	**Reijneveld, PNAS. 2020** **PMID: 32868441**
**CD1c**	**Vδ1**	**PBMC-sorted γδ T cells using** **CD1c tetramers loaded with microbial lipids**	**23-30 μM**	**Not reported**	**Roy, J Immunol 2016, PMID: 26755823**
**CD1d**	**V81**	**PBMC-sorted γδ T cells using CD1d tetramers**	**16-33 μM**	**resolved**	**Luoma, Immunity, 2013** **PMID: 24239091** **Uldrich, Nat Immunol, 2013 PMID: 24076636**
**EPCR**	**Vδ5/Vγ4**	**Clonally expanded γδ T cells in** **the context of CMV-infection**	**90 μM**	**Not reported**	**Willcox, Nat Immunol,** **2012 PMID: 22885985**
**MR1**	**Vδ3/Vγδ Vδ1/Vγ9**	**PBMC-sorted γδ T cells using MR1 tetramers**	**0.6-13 μM**	**resolved**	**Le Nours, Science, 2019, PMID: 31857486 Rice, PNAS, 2021, PMID:** **34845016**
**HLA-A2/MART-1**	**Vδ1/Vγδ**	**γδ T cells differentiated *in vitro*** **from HSPC**	**3-71 μM**	**resolved**	**Benveniste, Sci Immunol, 2018 PMID:** **30552102**
**HLA-DR**	**Vδ1/Vγ3**	**Clonally expanded γδ T cells in** **the context of CMV-infection**	**3-32 μM**	**Not reported**	**Deseke, J Exp Med,** **2022 PMID: 35852466**
**Others**					
**Annexin A2**	**Vδ3/Vγ8**	**PBMC γδ T cells reactive** **against lymphoma B cells *in vitro***	**3 μM**	**Not reported**	**Marlin, PNAS, 2017** **PMID: 28270598**
**BTNL3**	**Vγ4**	**Intestinal γδ T cells** **CMV expanded Vγ4Vδ1 T cells**	**20 μM**	**Not reported**	**Melandri, Nat Immunol, 2018, PMID: 30420626** **Willcox, Immunity, 2019,** **PMID: 31628053**
**BTN2A1**	**Vδ2/Vγ9 Vδ1/Vγ9**	**Blood Vγ9Vδ2 T cells, CMV-expanded or BTN2A1 tetramer-sorted Vγ9Vδ1 T cells**	**40-50 μM**	**Not reported**	**Rigau, Science, 2020,** **PMID: 31919129** **Karunakaran, Immunity, 2020, PMID: 32155411**

**Table 3:** Examples of ongoing γδ T cell-based clinical trials in cancer

		Approach	Clinical trial(s)	Institution/ Company	Therapeutic (product)	Cancer indications
			NCT04165941, NCT05664243	U. Alabama, IN8Bio	Chemotherapy-resistant allogeneic or autologous expanded γδ T cells (DeltEX)	Glioblastoma
			NCT03533816	U. Kansas, IN8Bio	Allogeneic expanded γδ T cells (EAGD) post-HSCT	Leukemias and myelodysplastic syndromes
			NCT05886491	Takeda	Allogeneic expanded Vδ1 T cells (TAK012)	Relapsed /refractory (r/r) AML
		Unmodified ACT	NCT05358808	TC Biopharm	Allogeneic expanded γδ T cells (TCB-008)	r/r AML
			NCT05015426	Lee Moffit Cancer Center	Allogeneic expanded γδ T cells (AAPC)	AML
			NCT05400603	Emory University	Allogeneic expanded γδ T cells	r/r Neuroblastoma
			NCT03183206, NCT03183219, NCT03183232, NCT03180437	Fuda Cancer Hospital affiliated with Jinan University (Guangzhou)	Allogeneic expanded Vγ9Vδ2 T cells	late-stage lung and liver cancer
			NCT04696705	Beijing GD Initiative Cell Therapy Technology	Allogeneic expanded γδ T cells	Non-Hodgkin lymphoma, peripheral T cell lymphoma
			NCT04764513	Chinese PLA General Hospital	Allogeneic expanded γδ T cells	AML, ALL, myelodysplastic syndromes and lymphoma
			NCT04765462	Chinese PLA General Hospital	Expanded allogeneic γδ T cells	Various solid tumours
			NCT06069570	Kiromic BioPharma	Allogeneic expanded γδ T cells combined with radiotherapy	Metastatic Non-Small Cell Lung Cancer
		CAR-ACT	NCT05546723	Luminary	BAFF-transduced Vδ1+Vδ2 T cells (LMY-920)	r/r Multiple myeloma
			NCT04735471, NCT04911478	Adicet	CD20-specific CAR-transduced Vδ1 T cells (ADI-001)	B cell lymphomas
			NCT05302037	Cytomed	NKG2D ligand-specific CAR-transduced Vγ9Vδ2 T cells (CTM-N2D)	Solid and hematological tumors
			NCT06193486	Lee Moffitt Cancer Center	PSCA-specific CAR-transduced γδ T cells	Metastatic Castration Resistant (mcr) Prostate Cancer
			NCT06150885	Ever Supreme Biotech	HLA-G-specific CAR-transduced γδ T cells	r/r solid tumours
		Conjugated ACT	NCT05653271	Acepodia	Anti-CD20 conjugated Vδ2 T cells (ACE1831)	Non-Hodgkin’s Lymphoma
			NCT06415487	Acepodia	Ant-EGFR conjugated Vδ2 cells (ACE2016)	Solid tumours
		γδTCR-ACT	NCT04688853	Gadeta	aβ T cells transduced with Vγ9Vδ2 TCR (TEG-002)	r /r multiple myeloma
			NCT04014894	Eureka	CD19-specific antibody/ γδTCR-transduced T cells (ET019003)	B cell lymphoma
			NCT04864054	Eureka	GPC3-specific antibody/ γδTCR transduced T cells (ECT204)	Liver cancers
			NCT04502082; NCT04634357	Eureka	Alpha-fetoprotein-specific antibody/ γδTCR transduced T cells (ET140203)	Liver cancers
		Engagers	NCT04243499, NCT05307874	ImCheck	BTN3A agonist (ICT01)	Hematological and solid tumors
			NCT05369000	Lava	PSMA-targeting bispecific γδ T Cell engager (LAVA-1207)	mcr Prostate Cancer
					
